# Sulfur-Doped Carbon Nitride Polymers for Photocatalytic Degradation of Organic Pollutant and Reduction of Cr(VI)

**DOI:** 10.3390/molecules22040572

**Published:** 2017-04-01

**Authors:** Yun Zheng, Zihao Yu, Feng Lin, Fangsong Guo, Khalid A. Alamry, Layla A. Taib, Abdullah M. Asiri, Xinchen Wang

**Affiliations:** 1 State Key Laboratory of Photocatalysis on Energy and Environment, College of Chemistry, Fuzhou University, Fuzhou 350002, China; zhengyun1101@163.com (Y.Z.); yuzihao15@163.com (Z.Y.); linfeng13@mails.ucas.ac.cn (F.L.); guofangsong@outlook.com (F.G.); 2 Chemistry Department, Faculty of Science, King Abdulaziz University, Jeddah 21589, Saudi Arabia; k_alamry@yahoo.com (K.A.A.); laylataib@gmail.com (L.A.T.); aasiri2@kau.edu.sa (A.M.A.); 3 Center of Excellence for Advanced Materials Research (CEAMR), Faculty of Science, King Abdulaziz University, Jeddah 21589, Saudi Arabia

**Keywords:** carbon nitride, self-assembly, photocatalysis, pollutant degradation, Cr(VI) reduction

## Abstract

As a promising conjugated polymer, binary carbon nitride has attracted extensive attention as a metal-free and visible-light-responsive photocatalyst in the area of photon-involving purification of water and air. Herein, we report sulfur-doped polymeric carbon nitride microrods that are synthesized through thermal polymerization based on trithiocyanuric acid and melamine (TM) supramolecular aggregates. By tuning the polymerization temperature, a series of sulfur-doped carbon nitride microrods are prepared. The degradation of Rhodamine B (RhB) and the reduction of hexavalent chromium Cr(VI) are selected as probe reactions to evaluate the photocatalytic activities. Results show that increasing pyrolysis temperature leads to a large specific surface area, strong visible-light absorption, and accelerated electron-hole separation. Compared to bulk carbon nitride, the highly porous sulfur-doped carbon nitride microrods fabricated at 650 °C exhibit remarkably higher photocatalytic activity for degradation of RhB and reduction of Cr(VI). This work highlights the importance of self-assembly approach and temperature-control strategy in the synthesis of photoactive materials for environmental remediation.

## 1. Introduction

With the rapid advancement of urbanization and industrialization, a series of environmental issues has come about owing to the excessive industrial contaminations containing toxic organic pollutants and poisonous metal ions [1]. Semiconductor-mediated photocatalysis technology has been regarded as the most promising strategy for environmental treatment of pollutants and heavy metal ions [2,3]. To date, various semiconductors, such as metal oxides, nitrides and sulphides, have been developed and further utilized as photocatalysts [4,5,6]. However, the low quantum efficiency, expensive raw materials and activity instability raise the threshold of practical applications of photocatalytic technology. Therefore, the development of photocatalytically polymeric materials with abundant materials and ease modifications take more considerations.

Heptazine-based polymer melon, also denoted as graphitic carbon nitride (g-C_3_N_4_) for simplicity, has drawn significant attention in the past few years attributed to its unique chemical, electronic and (photo)catalytic properties [7,8]. g-C_3_N_4_ has been reported to be an attractive photocatalyst for water splitting, pollutant purification, CO_2_ reduction, organic synthesis, bacterial inactivation, etc. [9,10]. However, carbon nitride polymers synthesized via traditional routes usually possess limited surface area, moderate visible light absorption, high recombination rate of charge and carrier, and inefficient photocatalytic activity [11,12,13]. Recently, a variety of nanostructures have been created by the nanocasting methods [14,15], using traditional hard-templating approach to tune the texture and photocatalytic performance [16,17,18]. Meanwhile, soft-templating approaches are newly emerging, which endow the synthetic process with simple and the morphological tuning diversiform [19,20]. However, the molecules of soft templates are easily decomposed, which restrains the polycondensation of precursors during the thermal treatment and the structure optimization of carbon nitride. Therefore, the development of an effective pathway for the structure modification of polymeric carbon nitride is an urgent task. 

The supramolecular system based on small molecule self-assembly is a relatively new and fascinating area in material science [21,22,23]. For carbon nitride, thermal polymerization of hydrogen-bonded supramolecular aggregates stemming from trithiocyanuric acid-melamine (TM) and cyanuric acid-melamine complex have been synthesized [24,25,26,27,28]. By controlling the reaction conditions, the chemical and electron structure can be effectively tailored, and further affecting the photocatalytic activity of carbon nitride [29,30]. In addition, the supramolecular aggregates can be also modified via copolymerization, element doping, composite, heterojunction, and salt-melt method [31,32,33]. Such modification methods are usually relied on the additional chemicals (e.g., organic co-monomer, inorganic salt and acid) in the starting process, which may lead to an inhomogeneous mixture [34,35,36,37,38]. As far as we know, the utilization of a facile temperature-control protocol for the self-assembly synthesis of carbon nitride photocatalysts without adding extra additives is less explored.

In this work, by employing TM supramolecular aggregates as the starting material, nanostructured carbon nitride is prepared via direct thermal condensation in Ar flow. The pre-organization of trithiocyanuric acid and melamine at molecular level intrinsically tunes the microstructure and morphology of target materials. By controlling the condensation temperature of TM, a series of sulfur-doped carbon nitride microrods with diverse properties and photocatalytic activities are obtained. The effects of pyrolysis temperature on the condensation degree, morphology and photocatalytic performances of the resultant carbon nitride are analyzed. In addition, the photocatalytic activity tests and the corresponding mechanism about dyes decomposing and Cr(VI) reduction are also carried out and discussed. Our findings will open up new opportunities for future design and development of polymeric materials for environmental remediation and energy conversion.

## 2. Results and Discussion

### 2.1. Characterization of TM Supramolecular Aggregates 

**Scheme 1:**
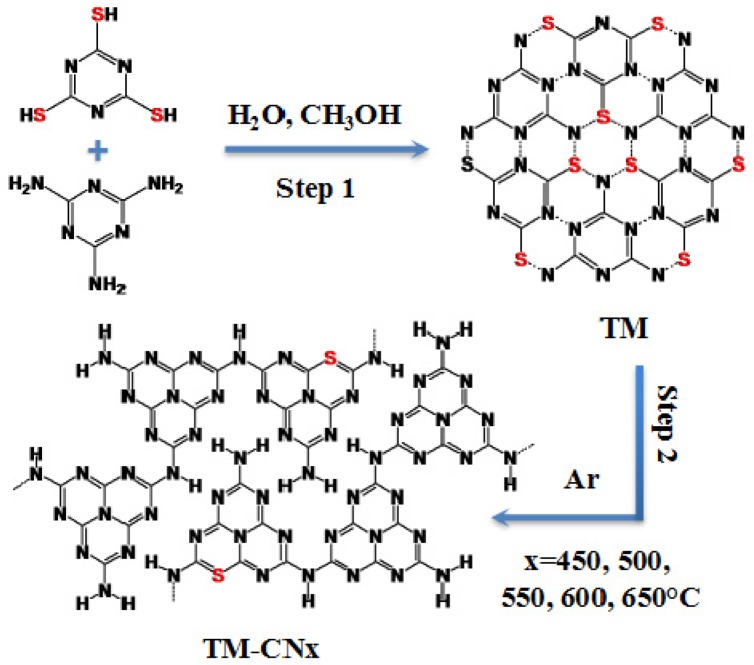
Schematic illustration of the formation process of TM-CNx.

**Figure 1:**
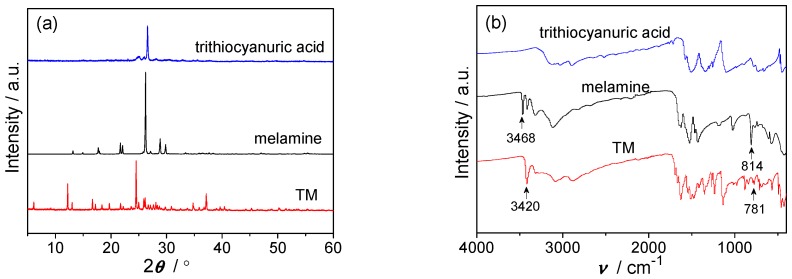
(**a**) XRD patterns and (**b**) FT-IR spectra of trithiocyanuric acid, melamine and TM.

TM supramolecular aggregate is synthesized from a simplified way by mixing an equimolar mixture of trithiocyanuric acid and melamine in CH_3_OH/H_2_O solution at room temperature (Scheme 1), which is different from hydrothermal method in the previous literature [39,40,41,42]. As shown in Figure 1a, TM cocrystal exhibits a totally different X-ray diffraction (XRD) pattern compared with pristine raw materials. The peaks at 12.3°, 13.1°, and 18.4° are ascribed to the in-planar packing, while the peak at 24.6° with a *d*-spacing of 0.362 nm is attributed to graphite-like stacking of individual two-dimensional sheets [40,41,42]. Fourier transform infrared spectroscopy (FT-IR) spectra (Figure 1b) indicate that the functional groups of both trithiocyanuric acid and melamine are retained in the TM adduct. Additionally, benefiting from the hydrogen-bonding interaction between trithiocyanuric acid and melamine, the triazine ring vibration of melamine is shifted to a lower wave-number from 814 to 781 cm^−1^, and the N–H stretching vibration of melamine is shifted to a lower wave-number from 3468 to 3420 cm^−1^ with enhanced intensity [40,41,42]. These results confirmed that hydrogen-bonded TM supramolecular aggregates are formed. 

### 2.2. Morphology of TM-CNx

The TM supramolecular aggregates are then heated at various temperature (450, 500, 550, 600, and 650 °C) in Ar gas to induce polymerization. The resultant carbon nitride products are abbreviated as TM-CNx, where x is the thermal polymerization temperature. As the condensation temperature rose (450 to 650 °C), the yields of products were decreased. It is worth noting that almost no product can be obtained at the condensation temperature of 700 °C due to the complete decomposition of TM. 

**Figure 2:**
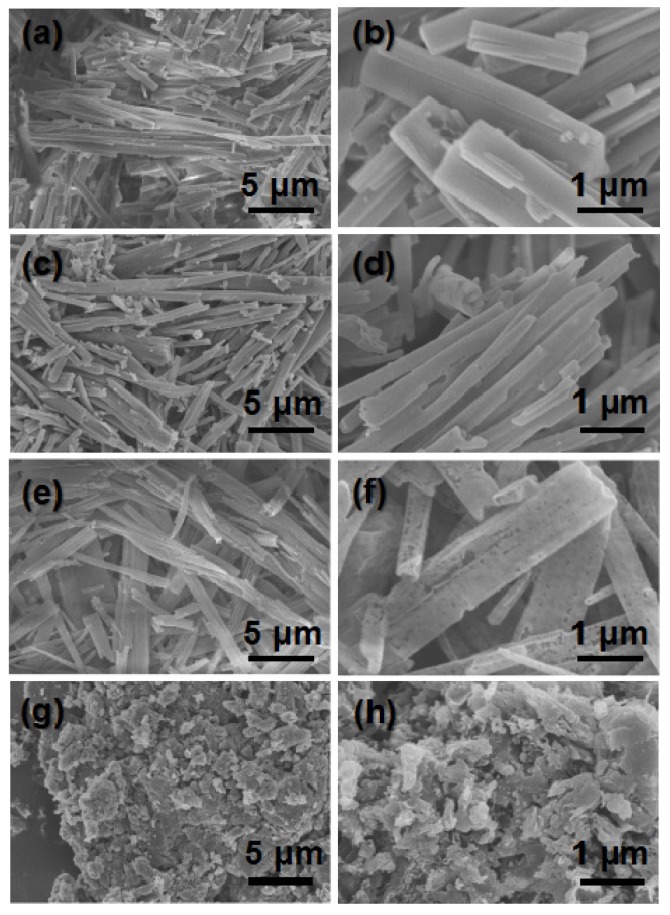
SEM images of (**a**,**b**) TM, (**c**,**d**) TM-CN550, (**e**,**f**) TM-CN650 and (**g**,**h**) bulk g-C_3_N_4_.

**Figure 3:**
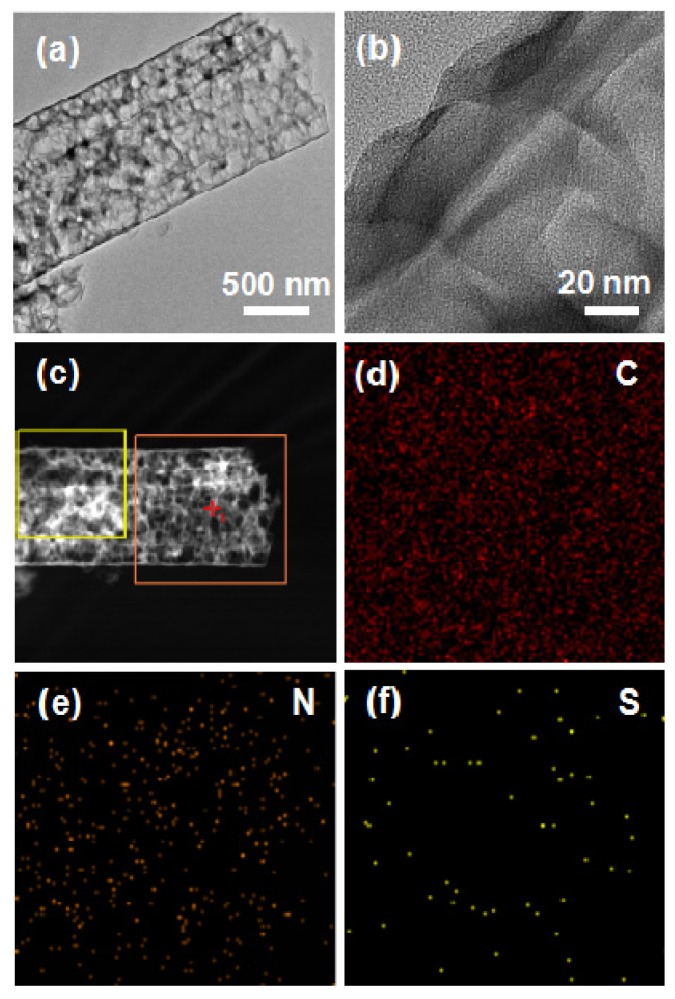
(**a**,**b**) TEM images and (**c**–**f**) elemental mapping images of TM-CN650.

To analyze the morphology of the as-prepared samples, the characterizations including scanning electron microscopy (SEM) and transmission electron microscopy (TEM) were performed. Figure 2 shows a smooth rod-like morphology for TM aggregates with the average width and length of 1 μm and 5–10 μm, respectively. After pyrolysis, the TM-CN550 sample preserves integrated rod morphology, whereas the TM-CN650 sample possesses a loose rod-like appearance with abundant pores and channels. The variation of nanostructure from TM to TM-CNx can be attributed to the thermal-driven rearrangement of some atoms as well as the breaking and reformation of some chemical bonds in TM precursor. Increasing the temperature from 550 °C to 650 °C leads to more pores due to the sulfur species elimination. TEM images of TM-CN650 (Figure 3) reveal a microrod morphology composed of spatially interconnected nanosheets and enormous macrospores. The elemental-mapping image of TM-CN650 shows that two major elements of C and N (as well as trace amount of S) are homogeneously distributed over the entire nanoarchitecture. 

### 2.3. Texture and Chemical Structure of TM-CNx

**Figure 4:**
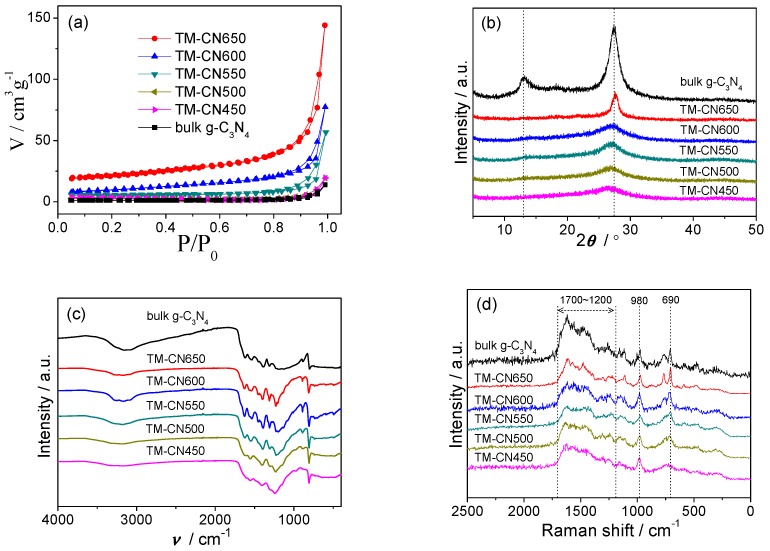
(**a**) N_2_ adsorption-desorption isotherms, (**b**) XRD patterns, (**c**) FT-IR spectra and (**d**) Raman spectra of TM-CNx and bulk g-C_3_N_4_.

The porous structure and surface area of TM-CN650 were studied by N_2_ sorption measurements (Figure 4a and Table 1). A characteristic type-IV isotherm with an H3 hysteresis loop was observed in the N_2_ sorption isotherms of TM-CN650, suggesting that the macroporous structure is formed by the accumulation of carbon nitride sheet. It indicates that TM-CN650 has a Brunauer–Emmett–Teller (BET) surface area of 72 m^2^ g^−1^, which is much higher than that of bulk g-C_3_N_4_ (3 m^2^ g^−1^) and other TM-CNx synthesized at lower temperatures. When the temperature is decreased from 650 to 450 °C, the BET surface area of TM-CNx is remarkably reduced, and the pore volume is lowered. These results clearly proved that the variation of temperature greatly changes the texture of carbon nitride. 

The crystal and chemical structure of the as-prepared TM-CNx polymers were carefully investigated by XRD, FT-IR, UV-Raman, and X-ray photoelectron spectroscopy (XPS) measurements. As shown in Figure 4b, all TM-CNx samples presented similar XRD patterns, which is an indication of heptazine-based polymeric melon. The characteristic diffraction peaks of TM-CNx at ca. 27° are assigned to the periodic in-plane tri-*s*-triazine stacking and the interlayer structural aromatic packing, and also ascribed to the (002) plane of the graphitic layer structures. As reaction temperature increases from 450 °C to 650 °C, the interlayer stacking peaks of TM-CNx samples shift from 26.6° to 27.4°, corresponding to the decrease of interlayer distance from 0.335 to 0.326 nm. This phenomenon suggests an improvement of crystallinity and a decreased interlayer distance, thus proving an enhanced condensation degree and more regular stacking structure of carbon nitride with increasing heating temperature [39]. Compared to typical XRD pattern of bulk g-C_3_N_4_ material, these TM-CNx samples exhibit relatively weaker intensities at 27°, and the peak at ca. 13° as the (100) peak is not evident in TM-CNx samples. This result suggests the reduced order degree of chemical structure owing to the incomplete molecular polymerization as well as the existence of one-dimensional nanostructure with increasing pyrolysis temperature.

FT-IR measurement (Figure 4c) was also performed to investigate the formation of carbon nitride. Both bulk g-C_3_N_4_ and TM-CNx polymers displayed similar FT-IR vibration modes. The peaks at 810 cm^−1^ and 1200–1600 cm^−1^ were assigned to the characteristic breathing and stretching vibration modes of aromatic C–N heterocycles, identifying the existence of triazine units [43]. The broad and weak bands at 2900–3300 cm^−1^ were typical signals of N–H or O–H vibrations, which were ascribed to the uncondensed amino groups as the surface terminal groups and the absorbed H_2_O molecules. 

To probe the chemical structure of TM-CNx, Raman spectra were carried out over the catalysts pressed on glass slides (Figure 4d). With a 325 nm UV laser excitation, an intense, broad, asymmetric peak in the range of 1200–1700 cm^−1^ can be ascribed to C–N stretching vibrations, which resembles the “G” and “D” band profiles for structurally disordered graphitic carbon-based materials. The existence of a heptazine ring structure is further confirmed by the two sharp peaks appeared at 690 and 980 cm^−1^ [44]. The former peak at 690 cm^−1^ is a doubly degenerate mode for in-plane bending vibrations of the heptazine linkages, while the latter peak at 980 cm^−1^ is assigned to the symmetric N-breathing mode of heptazine units. No band is observed between 2000 and 2500 cm^−1^, since there is no triply bonded C≡N units or N=C=N groups within the carbon nitride framework. These features are observed for all of the TM-CNx catalysts.

**Figure 5:**
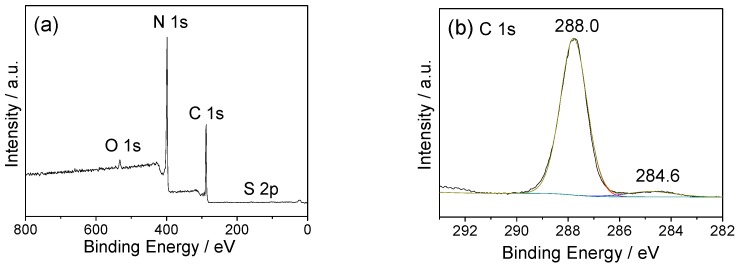

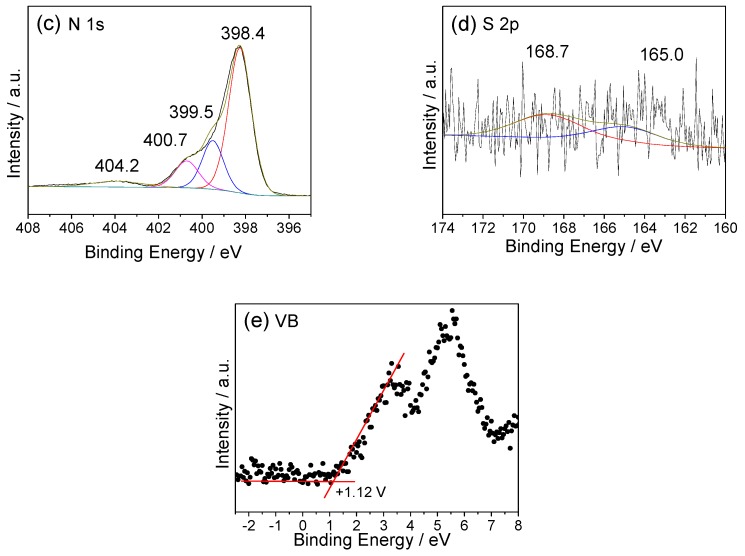
(**a**) XPS survey spectra, (**b**) C 1s spectra, (**c**) N 1s spectra, (**d**) S 2p spectra, (**e**) VB XPS spectra of TM-CN650.

Additional proof for the formation of polymeric carbon nitride was obtained by XPS measurements of TM-CN650 (Figure 5). The weak peak at 284.6 eV was attributed to the sp^2^-hybridized carbon (C–C) of standard carbon [43]. The strong peak at binding energy of 288.0 eV was determined as the sp^2^-bonded carbon atoms in the heterocycle (N=C–N) of aromatic carbon nitride, which was related to the major skeleton carbon in the triazine-based heterocycle [43]. The high resolution of N 1s spectra could be deconvoluted into four peaks at 398.4, 399.5, 400.7 and 404.2 eV, respectively [43]. These peaks were corresponded to the sp^2^ bonded nitrogen (C–N=C), the tertiary nitrogen (N–C_3_) groups, the surface uncondensed amino groups (C–N–H), and the charging effects or positive charge localization in the heterocycles, respectively [43]. The first two nitrogen together with the sp^2^-bonded carbon (N–C=N) constitute the triazine-based heterocyclic ring (C_6_N_7_) units [43]. Weak signal of S 2p can be detected in TM-CN650 sample, proving the existence of sulfur species in carbon nitride structure. The weak peak of S 2p at 165.0 eV is ascribed to the generation of S-N bonds by replacing lattice carbon with sulfur, while the peak at 168.2 eV is determined as the formation of sulfur oxide during the thermal condensation. According to the XPS spectra of TM-CN650, the atomic concentration ratio of S/C is calculated to be 1:1627 based on the method reported by Huang et al. [45]. Thus, it can be concluded that sulfur has been doped into the structure of carbon nitride. 

To further confirm the element composition of TM-CNx, the elemental analysis was performed. As shown by the elemental analysis result (Table 1), the C/N molar ratios of TM-CNx under different temperatures remain in the range of 0.66–0.69 without any major fluctuations, similar to that of bulk g-C_3_N_4_ (0.68). All samples feature 2 wt. % hydrogen (Table 1), suggesting that these materials are indeed polymeric melon rather than fully-condensed phase of covalent carbon nitride materials. Tiny sulfur can be found in TM-CNx. It can be concluded from these results that the structure of TM-CNx are sulfur-doped and heptazine-based melon polymers.

### 2.4. Optical Property and Band Structure of TM-CNx

**Figure 6:**
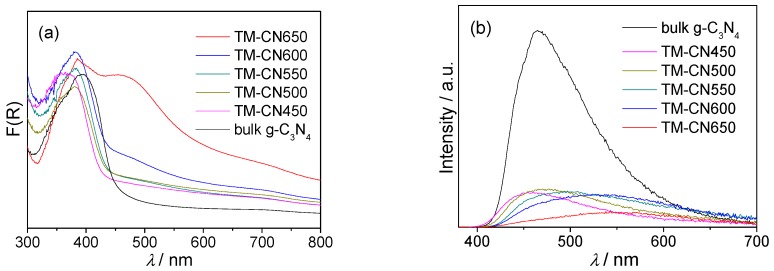
(**a**) UV-Vis DRS spectra and (**b**) PL spectra of TM-CNx samples.

The optical properties of the TM-CNx samples were investigated by UV-Vis diffuse reflectance (DRS) spectra (Figure 6a and Table 2). All TM-CNx samples feature typical semiconductor-like absorption. Considerable improved light-harvesting capability and gradual bathochromic shift of optical absorption edges are found for TM-CNx samples prepared under increasing condensation temperature. By incresing the condensation temperature, the absorption band edges of TM-CNx samples red-shifted from 442 to 680 nm, and the electronic band gap narrowed from 2.81 to 1.82 eV for TM-CN450 to TM-CN650. The modified light-absorption property from temperature processing is primarily attributed to the emergence of structural distortion and the activation of more n→π* transitions, as well as the improved π-electron delocalization and inter-planar packing towards J-type aggregates in the conjugated system [39]. The increase of visible light absorption ability and the decrease of band gap contributed to the capturing of more visible photons, which is beneficial for enhancing the photocatalytic activity. Combined with the band gap of 1.82 eV and the valence-band (VB) potential of 1.12 eV (determined by the VB XPS spectra in Figure 5e), the conduction band (CB) level of TM-CN650 is calculated to be −0.70 eV. 

The charge-carriers separation/recombination rates were next investigated by room temperature photoluminescence (PL) spectra under excitation wavelength of 370 nm (Figure 6b). All the fluorescence of TM-CNx are quenched in comparison with bulk g-C_3_N_4_, indicating the lower exciton energy and the improved electron-hole separation in TM-CNx. Additionally, the PL intensities of the emission peaks were greatly decreased when the temperature were increased, which illustrates that raising temperature is effective for suppressing the rapid charge carrier recombination. The quenching of emission intensity also suggests that the relaxation of a portion of photocarriers occurs via a non-radiative pathway, presumably due to charge transfer of electrons and holes to new localized/surface states [46]. Moreover, when the temperature is varied from 450 to 650 °C, an obvious red-shift of the emission peak from 460 to 560 nm was observed for TM-CNx, which further certified the decreased band-gap energy of TM-CNx samples.

### 2.5. Photocatalytic Activity of TM-CNx in Degradation of RhB

**Figure 7:**
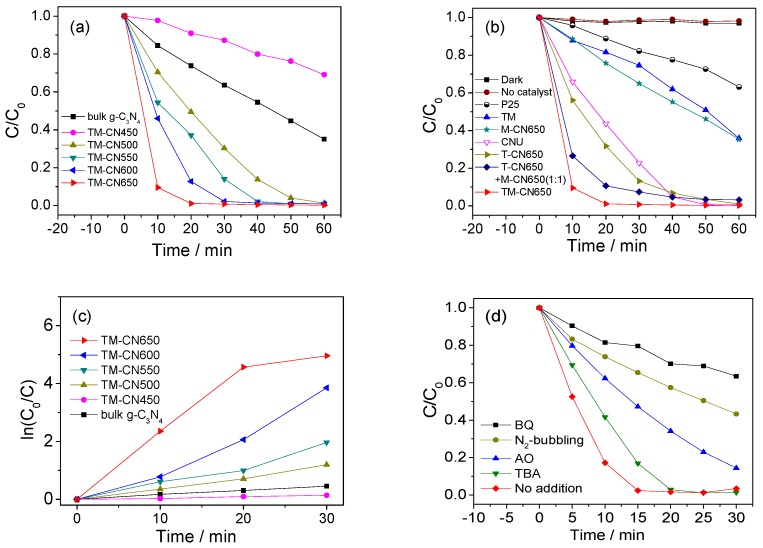

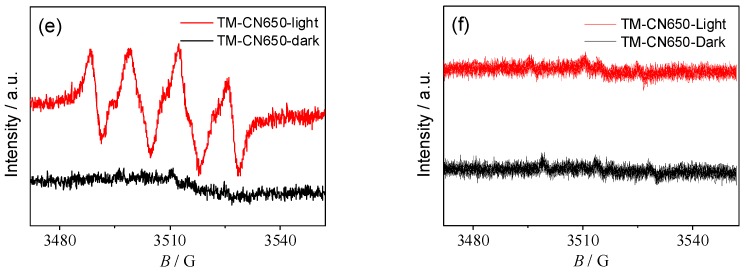
(**a**,**b**) Concentration changes of RhB as a function of irradiation time with different catalyst under visible light irradiation; (**c**) first-order rate constant *k* (min^−1^) of TM-CNx; (**d**) effect of quencher additive and N_2_ purging on the photocatalytic activity of TM-CN650 in RhB degradation. DMPO spin-trapping EPR spectra of TM-CN650 sample with visible light irradiation (λ > 400 nm) for the detection of (**e**) DMPO-·O_2_^−^ and (**f**) DMPO-·OH.

The photocatalytic activities of the TM-CNx samples were evaluated by degradation of Rhodamine B (RhB) under visible light (λ > 420 nm) irradiation. The photodegradation process was recorded by the temporal evolution of the spectra of supernants at different reaction times. Figure 7 displays the changes of the RhB concentration versus the reaction time over the TM-CNx catalysts and the corresponding first-order kinetics plot by the equation of ln(C_0_/C) = *k*t, where C_0_ and C are the RhB concentrations in solution at times 0 and t, respectively, and *k* is the apparent first-order rate constant. Based on the result of control experiments, RhB was not degraded under dark conditions, and RhB is also stable under visible light if there is no photocatalyst involved. As shown in Figure 7a, the TM-CNx samples exhibit accelerated degradation ability and higher photocatalytic activity with increasing condensation temperature. An optimal activity was found for TM-CN650 with the superior degradation rate of 97% after 15 min. Furthermore, TM-CN650 shows much better photocatalytic activity than bulk g-C_3_N_4_, urea derived carbon nitride (CNU), and commercial Degussa P25 (Figure 7b). Figure 7c and Table 2 show the first-order rate constant *k* (min^−1^) measurements for RhB degradation of the TM-CNx samples. The measured *k* value of TM-CN650 is 0.2283 min^−1^, which is almost 14 times higher than that of bulk g-C_3_N_4_ (0.0152 min^−1^). 

**Figure 8:**
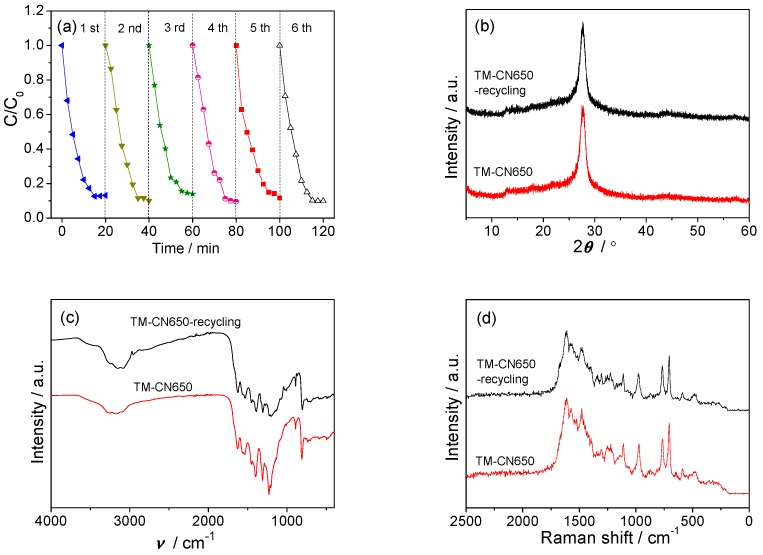
(**a**) cycling runs of RhB degradation in TM-CNx under visible light irradiation; (**b**) XRD patterns, (**c**) FT-IR spectra, and (**d**) Raman spectra of TM-CN650 and recycled TM-CN650 after photocatalytic degradation.

The stability of TM-CN650 photocatalyst was investigated by recycling the photocatalyst for RhB degradation under visible-light irradiation (Figure 8). The high degradation capability of TM-CN650 was maintained without a significant decrease in six consecutive experiments. The XRD, FT-IR, and Raman analysis also proved that the crystal and chemical structure of TM-CN650 remain unchanged after the photocatalytic reaction. These results proved the good stability of TM-CN650 in the photocatalytic reaction. 

To further investigate the possible photodegradation mechanism, the effects of various radical scavengers and N_2_ purging on the degradation of RhB over TM-CN650 were examined (Figure 7d). The photodegradation of RhB was repeated with modification by adding benzoquinone (BQ), tert-butyl alcohol (TBA), and ammonium oxalate (AO) as the scavengers of superoxide radical (·O_2_^−^), hydroxyl radical (·OH) and hole (h^+^), respectively. To suppress the capture of photo-induced electrons by oxygen to generate ·O_2_^−^, N_2_ bubbling was also applied to remove the oxygen molecules dissolved in the RhB aqueous solution. After 15 min of reaction, the removal efficiency of RhB are 20%, 34%, 53% and 83% in the presence of BQ, N_2_ bubbling, AO and TBA, respectively. It was observed that the degradation rate of RhB was depressed under an N_2_ atmosphere. The addition of BQ almost completely hindered the decomposition of RhB. These results confirmed that ·O_2_^−^ was the major oxidation species during degradation of RhB in the TM-CN650/RhB system. The degrading rate of RhB also decreased obviously with the addition of AO, indicating that photogenerated hole oxidation plays a role in the degradation of RhB. Additionally, the degradation rate was slightly reduced by the addition of TBA (an efficient trap of ·OH), which was important but did not result in complete quenching of the photodegradation reaction. The formation of reactive oxygen species over the TM-CN650 catalyst under visible light irradiation was further probed by a 5,5-dimethyl-1-pyrroline-*N*-oxide (DMPO) spin-trapping electron paramagnetic resonance (EPR) technique. DMPO/O_2_^−^ adducts were clearly observed when the TM-CN650 catalyst was exposed to visible light irradiation (Figure 7e), whereas no detectable ·OH signals can be observed in experiments carried out under darkness or visible light irradiation (Figure 7f). These results demonstrated the fact that ·OH is involved but not exclusively and that the major reactive species are ·O_2_^−^ and holes in RhB degradation with a TM-CN650 catalyst.

### 2.6. Photocatalytic Activity of TM-CNx in Reduction of Cr(VI)

**Figure 9:**
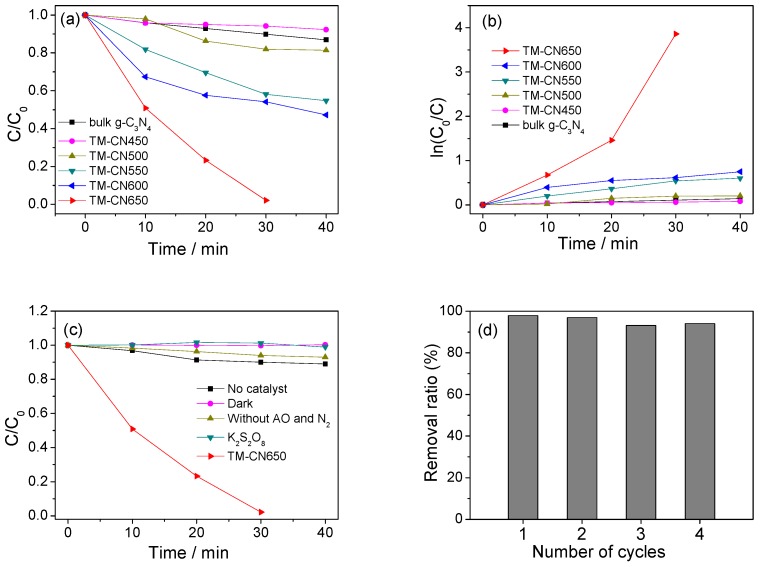
(**a**) concentration changes of Cr(VI) as a function of irradiation time with different catalyst under visible light irradiation; (**b**) first-order rate constant *k* (min^−1^) of TM-CNx in photocatalytic reduction of Cr(VI); (**c**) control experiments of photocatalytic reduction of Cr(VI); and (**d**) the reusability of TM-CN650 for the reduction of Cr(VI) after 40 min of irradiation.

In order to understand the visible light photocatalytic activity of the as-synthesized samples for Cr(VI) reduction, a test reaction was carried out for the reduction of 5 mg/L of Cr (VI) solution with the catalyst and ammonium oxalate (as hole scavenger) in nitrogen atmosphere under visible-light irradiation (λ > 400 nm). Figure 9 displays the changes of Cr (VI) concentration versus the reaction time over the TM-CNx catalysts. The apparent rate constant for the reduction of Cr(VI) is calculated by the following equation: *k* = ln(C_0_/C)/t, where C_0_ and C are the Cr (VI) concentrations in solution at times 0 and t, respectively, and *k* is the apparent first-order rate constant. As shown in Figure 9b and Table 2, the apparent reaction rate constant and photocatalytic activity of TM-CNx for reduction of Cr(VI) followed the order of TM-CN650 > TM-CN600 > TM-CN550 > TM-CN500 > bulk g-C_3_N_4_ > TM-CN450. Apparently, TM-CN650 exhibits the best photocatalytic activity in reduction of Cr(VI). Furthermore, the results of recycled experiment reveal that the TM-CN650 sample has good photostability, since there is no noticeable decrease in the removal ratio over four recycling tests (Figure 9d).

Some control experiments were performed to certify the possible mechanism of Cr(VI) reduction. As can be seen from Figure 9c, the reduction of Cr(VI) hardly occurs in the absence of light or photocatalyst. No significant reaction of Cr(VI) is observed without AO and N_2_, even when the reaction is operated in the presence of catalyst with irradiation. The reduction of Cr(VI) to Cr(III) by photogenerated electrons can be described by the following equation:

Cr_2_O_7_^2−^ + 14H^+^ + 6e^−^ → 2Cr(III) + 7H_2_O (1)

According to Equation (1), Cr (VI) was reduced to Cr(III) in the presence of TM-CN650 and AO upon purging with N_2_ under visible light irradiation. For the photocatalytic reduction of Cr(VI), AO as a hole scavenger can capture photo-induced holes (h^+^) of TM-CN650, and thus mitigate the recombination of photogenerated carriers of TM-CN650. Furthermore, the superoxide radicals (·O_2_^−^), which is formed by the transformation of photogenerated electrons to the absorbed O_2_, is significantly suppressed under N_2_ bubbling. Accordingly, the introduction of AO and purging with N_2_ atmosphere in the reaction solution play crucial roles in the photocatalytic reduction of Cr(VI). Additionally, the control experiments for the photoreduction of Cr(VI) under visible light illumination have been performed by using K_2_S_2_O_8_ as a scavenger for photogenerated electrons. A remarkably reduced activity was noticed by adding K_2_S_2_O_8_, further confirming that the reduction reaction of Cr(VI) is driven by the photoexcited electrons of TM-CN650 [47]. 

### 2.7. Mechanism of Photocatalytic Degradation of RhB and Reduction of Cr(VI) over TM-CN650

**Figure 10:**
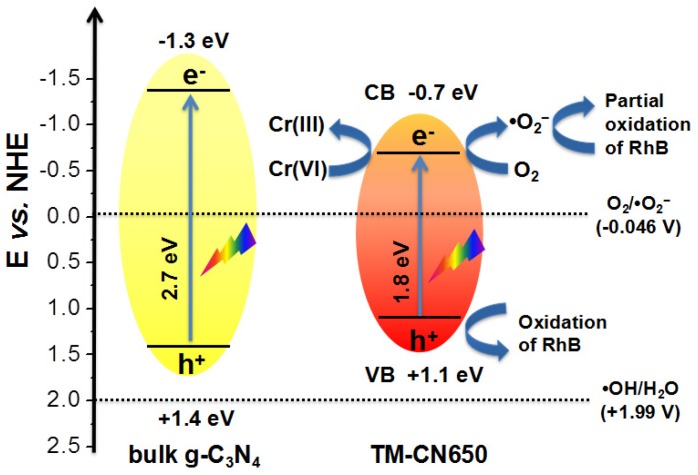
Schematic illustration of the mechanism of photocatalytic degradation of RhB and reduction of Cr(VI) over TM-CN650.

According to the above discussions, a possible mechanism for photocatalytic degradation of RhB and reduction of Cr(VI) by TM-CN650 is proposed and depicted in Figure 10. With visible light irradiation, a photon is absorbed by the TM-CN650 semiconductor, and an electron is excited from the VB to the CB, generating a positive hole in the VB and an electron in the CB. The CB potential (−0.7 eV) of TM-CN650 is negative enough to E(O_2_/·O_2_^−^) (−0.046 eV vs. normal hydrogen electrode (NHE)) [48,49], and O_2_ adsorbed on the surface of TM-CN650 can be reduced to ·O_2_^−^ by the electrons left in the CB via one electron reducing reaction. It is noted that ·O_2_^−^ is a relatively mild oxidant that results in partial oxidation (the cleavage of the RhB chromophore structure) instead of mineralization [50,51]. According to the VB potential (+1.1 eV) of TM-CN650, hydroxyl groups or water molecules adsorbed on the surface of TM-CN650 can not be directly oxidized to ·OH radicals (+1.99 eV vs. NHE). The generation of ·OH via photogenerated electron-induced multistep reduction of O_2_ (O_2_ + e→·O_2_^−^, ·O_2_^−^ + e^−^+ 2H^+^ → H_2_O_2_, H_2_O_2_ + e^−^→ ·OH + OH^−^) is involved but not exclusively in the current photocatalytic system [51,52]. The photodegradation of RhB by TM-CN650 catalyst is mainly attributed to the partial oxidation of RhB by ·O_2_^-^ and photogenerated hole oxidation of RhB. Different from the degradation of RhB, the photocatalytic reduction of Cr(VI) is directly reduced to Cr(III) by photo-generated electrons (e^−^) of TM-CN650. Benefiting from the one-dimensional nanostructure, high surface area, and appropriate band structure of TM-CN650, the recombination of photoinduced electron-hole pairs are inhibited and the lifetime of photo-induced charge carriers are prolonged, thereby contributing to the enhancement of overall photocatalytic activity in an aqueous phase [53,54,55]. 

## 3. Materials and Methods 

### 3.1. Chemicals

Melamine, Rhodamine B, ammonium oxalate, potassium dichromate (K_2_Cr_2_O_7_), potassium persulfate (K_2_S_2_O_8_), methanol, and tert-butyl alcohol were purchased from China Sinopharm Chemical Reagent Co. Ltd (Shanghai, China). Trithiocyanuric acid and benzoquinone were purchased from Aladdin Industrial Corporation (Shanghai, China). All of the chemicals were of analytical grade and were used without further purification. 

### 3.2. Synthesis of TM

The synthetic process was performed in a 250 mL beaker containing a 10 × 30 mm magnetic stirring bar. Trithiocyanuric acid (0.71 g, 4 mmol) and melamine (0.50 g, 4 mmol) were added with 120 mL deionized water and 25 L methanol. Then, the mixture was sonicated for 10 min, followed by stirring at 500 r for 10 min and at 250 r for 12 h. The as-obtained mixture was filtered to recover the solid precipitate, washed with deionized water (200 mL), and dried in an oven at 70 °C. 

### 3.3. Preparation of TM-CNx from TM

TM-CNx samples were synthesized by heating the TM precursor at a certain temperature (*x* = 450, 500, 550, 600, and 650) for 2 h with the heating rate of 4.6 °C/min under flowing Ar gas (200 mL/min). 

### 3.4. Preparation of Bulk g-C_3_N_4_

Bulk g-C_3_N_4_ sample was prepared by heating melamine (10 g) at 550 °C for 4 h with the heating rate of 2.3 °C/min in the air, and followed by grounding into power.

### 3.5. Preparation of CNU

Carbon nitride derived from urea (CNU) was prepared by heating urea (10 g) at 600 °C for 2 h with the heating rate of 4.6 °C/min in the air. 

### 3.6. Preparation of T-CN650 

T-CN650 sample was synthesized by heating trithiocyanuric acid (5 g) at 650 °C for 2 h with the heating rate of 4.6 °C/min under flowing Ar gas (200 mL/min). 

### 3.7. Preparation of M-CN650 

M-CN650 sample was synthesized by heating melamine (5 g) at 650 °C for 2 h with the heating rate of 4.6 °C/min under flowing Ar gas (200 mL/min). 

### 3.8. Characterization

XRD measurements were collected on a Bruker D8 Advance diffractometer (Billerica, MA, USA) with Cu Ka1 radiation (λ = 1.5406 Å). FT-IR spectra were collected with a thermo Nicolet Nexus 670 FT-IR spectrometer (Waltham, MA, USA), and the samples were mixed with KBr at a concentration of ca. 0.2 wt. %. UV-Raman scattering measurements were performed with a multichannel modular triple Raman system (Renishaw Co., Wotton-under-Edge, Gloucestershire, UK) with confocal microscope at room temperature using a 325 nm laser. XPS data were obtained on a Thermo Scientific ESCALAB250 instrument (Waltham, MA, USA) with a monochromatized Al Kα line source (200 W). All binding energies were referenced to the C 1s peak at 284.6 eV of surface adventitious carbon. Elemental analysis results were collected from Elementar Vario EL (Langenselbold, Germany). SEM measurement was conducted using Hitachi SU-8010 and S4800 Field Emission Scanning Electron Microscopes (Chiyoda, Tokyo, Japan). TEM measurement was obtained using a FEI TECNAIG2F20 instrument (Hillsboro, OR, USA). N_2_ adsorption-desorption isotherms were performed using a 3020 micromeritics tristarII surface area and porosity analyzer (Atlanta, GA, USA). The UV-Vis absorption spectra were measured on Shimadzu UV-1780 (Kyoto, Japan) and Varian Cary 50 UV/Vis spectrophotometer (Palo Alto, CA, USA). The UV-Vis DRS spectra were measured on Cary 5000 Scan UV-Vis-NIR system from Agilent Technologies (Santa Clara, CA, USA). Photoluminescence spectra were recorded on an Edinburgh FI/FSTCSPC 920 spectrophotometer (Livingston, West Lothian, UK). The EPR technique was used to detect ·O_2_^−^ or ·OH radicals spin-trapped by DMPO in methanol or water, respectively. The signals were collected by a Bruker model A300 spectrometer (Bruker Instruments, Inc., Billerica, MA, USA) with the settings of center field (3512 G), microwave frequency (9.86 GHz), and power (20 mW). 

### 3.9. Photocatalytic Degradation of RhB

An aqueous dispersion of catalyst and RhB dye was prepared for the photocatalytic test by dispersing 20 mg of catalyst powder to 80 mL RhB solution (10^−5^ mol/L). This reaction dispersion was magnetically stirred in the dark for ca. 30 min prior to irradiation to establish the adsorption/desorption equilibrium of the dye on the catalyst surface. The mixture was then irradiated under a 300 W Xe-lamp with cut-off filter to produce visible light irradiation (λ > 420 nm) at room temperature. At given irradiation time intervals, specimens (2 mL) were taken from the dispersion, and centrifuged at 10,000 rpm for 10 min to separate the catalyst particles. The concentration of aqueous RhB was determined using a Shimadzu UV-1780 UV-vis spectrophotometer at 554 nm by measuring its absorbance. The RhB degradation was calculated by the Lambert–Beer equation. Photoactivities for RhB in the dark in the presence of the photocatalyst and under visible-light irradiation in the absence of the photocatalyst were also evaluated.

### 3.10. Photocatalytic Activity for Reduction of Cr(VI)

In a typical measurement, a 300 W Xe arc lamp (PLS-SXE 300, Beijing Perfect Light Co., Ltd., Beijing, China) with a UV cut-off filter to eliminate light of wavelength λ < 400 nm was used as the light source. The power density of the light source used in the measurement is ca. 700 mW/cm^2^. In addition, 10 mg of the sample, 50 mg hole scavenger (ammonium oxalate), and 40 mL of the Cr(VI) aqueous solution (5 mg·L^−1^, which was based on Cr in a dilute K_2_Cr_2_O_7_ solution) were added in a quartz vial. After the photocatalyst was dispersed in the solution with an ultrasonic bath for 10 min, the above suspension was bubbled with nitrogen gas (100 mL/min) to remove air and was stirred in the dark for 1 h, in order to ensure the establishment of adsorption–desorption equilibrium between the sample and the reactant before being exposed to visible light illumination. During the process of the reaction, 4 mL of sample solution was collected at a certain time interval and centrifuged to remove the catalyst completely at 10,000 rpm for 10 min. The Cr(VI) content in the supernatant solution was analyzed colorimetrically at 540 nm using the diphenylcarbazide method [56,57] on a Varian UV-vis spectrophotometer (Cary-50, Varian Co., Palo Alto, CA, USA). 

## 4. Conclusions

In summary, one-dimensional sulfur-doped carbon nitride nanostructures have been prepared by thermal polymerization of trithiocyanuric acid-melamine supramolecular aggregates under Ar atmosphere at different temperatures. The acidic sulfur-containing gases generated from the thermal decomposition of precursors greatly affect the condensation/polymerization process, thus modifying both the texture and electronic structure of carbon nitride polymers [39,44]. Increasing the heating temperature not only causes a structure peeling effect to increase the porosity/surface area, but also induces structural distortion and the activation of more n→π* transitions to modify the band structure of carbon nitride polymers [39,44]. These integrative factors synergistically contribute to the overall photoactivity improvement of porous sulfur-doped carbon nitride microrods for degradation of RhB and reduction of Cr(VI). The porous rod-like sulfur-doped carbon nitrides synthesized at 650 °C demonstrates the best photocatalytic activity for degradation of RhB and reduction of Cr(VI), presumably owing to the unique electronic structure, strong light harvesting ability, high surface area, and accelerated separation rate of photo-induced electron-hole. Our findings not only significantly improve the understanding on the role of condensation temperature in the property of carbon nitride polymers, but also open the possibilities for the facile synthesis of nanostructured materials to manipulate their activity for photon-involving purification of water and air. 

## Figures and Tables

**Table 1:** The surface area, pore volume, and elemental composition of TM-CNx samples.

Entry	Samples	Surface Area (m^2^ g^−1^)	Pore Volume (cm^3^ g^−1^)	C (wt. %)	N (wt. %)	H (wt. %)	S (wt. %)	Molar ratio C/N (%)
1	bulk g-C_3_N_4_	3	0.01	34.1	58.6	2.0	-	0.68
2	TM-CN450	8	0.01	32.1	56.4	2.3	＜0.5	0.66
3	TM-CN500	9	0.01	33.1	57.3	2.1	＜0.5	0.67
4	TM-CN550	17	0.01	33.4	57.1	2.3	＜0.5	0.68
5	TM-CN600	35	0.04	32.8	55.6	2.2	＜0.5	0.69
6	TM-CN650	72	0.06	32.8	55.8	2.2	＜0.5	0.69

**Table 2:** The absorption band edge, band gap energy, and photocatalytic activity of TM-CNx samples.

Entry	Samples	Absorption Band Edge (nm)	Band Gap Energy (eV)	*k_RhB_* (min^−1^)	*k_Cr__(VI)_* (min^−1^)
1	bulk g-C_3_N_4_	460	2.70	0.0152	0.0036
2	TM-CN450	442	2.81	0.0047	0.0020
3	TM-CN500	452	2.74	0.0352	0.0066
4	TM-CN550	456	2.72	0.0496	0.0181
5	TM-CN600	463	2.68	0.1031	0.0204
6	TM-CN650	680	1.82	0.2283	0.1287
